# Pyloric Stenosis in a Patient with CEDNIK Syndrome

**DOI:** 10.7759/cureus.59475

**Published:** 2024-05-01

**Authors:** Mark A Potesta, Vivian Aldana, Samit Patel

**Affiliations:** 1 Medical School, Lake Erie College of Osteopathic Medicine, Bradenton, USA; 2 Pediatrics, BayCare St. Joseph’s Children’s Hospital, Tampa, USA; 3 Gastroenterology, BayCare St. Joseph’s Children’s Hospital, Tampa, USA; 4 Gastroenterology, Pediatric Gastroenterology and Nutrition of Tampa Bay, Tampa, USA; 5 Pediatrics, Lake Erie College of Osteopathic Medicine, Bradenton, USA

**Keywords:** pachygyria, gastric outlet obstruction, rare genetic diseases, pyloric stenosis, cednik syndrome

## Abstract

We present a rare neurocutaneous genetic disorder where patients develop a combination of cerebral dysgenesis, neuropathy, ichthyosis, and keratoderma, commonly known as CEDNIK syndrome. It is an autosomal recessive inheritance involving the SNAP29 protein, mapped to the 22q11.2 gene. Phenotypic variation is seen with this disease, with clinical manifestation of developmental milestone delays ranging in severity. With only a handful of documented cases, available research, management of the syndrome, and prognosis are not well established. As CEDNIK syndrome has systemic implications, care coordination between specialists is essential in improving patient outcomes. Particularly important is preventing patients from meeting the criteria of failure to thrive, a commonly reported issue. In this case, we present a four-month-old male with a past medical history of pyloric stenosis status/post pyloromyotomy who has failure to thrive, gastroesophageal reflux disease, profound hypotonia, and delayed progression of developmental milestones. Additionally, the case is complicated by idiopathic pyloric stenosis, further contributing to the patient’s failure to thrive. We aim to discuss the pathophysiology of this syndrome, explore the timeline of disease progression, as well as compare our case to the current literature.

## Introduction

A rare neurocutaneous syndrome classified by a constellation of symptoms pertaining to cerebral dysgenesis, neuropathy, ichthyosis, and keratoderma was first described in 2005 [1]. The collective nature of these symptoms comprises a rare diagnosis known as CEDNIK syndrome. In 2021, the prevalence of this syndrome was only 19 patients worldwide [2]. Of note, these 19 patients descend from 10 unrelated families [2]. This autosomal recessive syndrome results from loss of function variations in the synaptosomal associated protein 29 (SNAP29) protein [2]. The SNAP29 protein codes for a SNARE protein, which is involved in vesicular transport and fusion [3]. As first reported in 2005, the SNAP29 protein is mapped to the 22q11.2 gene [3]. Phenotypic variation is seen in this condition, with disease manifestation encompassing psychomotor slowing, global developmental delay, profound hypotonia, roving eye movements, failure to thrive, progressive microcephaly, facial dysmorphism, visual impairment, sensorineural hearing loss, ichthyosis, and palmoplantar keratoderma [1, 2]. As a result of the profound hypotonia, patients often fail to meet appropriate developmental milestones, suffering from feeding difficulties leading to failure to thrive [2]. The profound rarity of this syndrome has contributed to many unanswered questions pertaining to treatment and prognosis.

## Case presentation

On initial presentation, a two-month-old male with a past medical history of pyloric stenosis status/post pyloromyotomy presented to the gastroenterology clinic with failure to thrive, gastroesophageal reflux disease (GERD), and stridor. He was born via cesarean section in a breech position at 40 weeks gestation. The patient experienced no postnatal complications, with a birth weight of 6 lbs 13 oz. In the clinic, he appeared emaciated and tachypneic with subcostal retractions and tracheal tugging. His respiratory rate was greater than 60, and he was less than the first percentile weight for his age.

**Figure 1:**
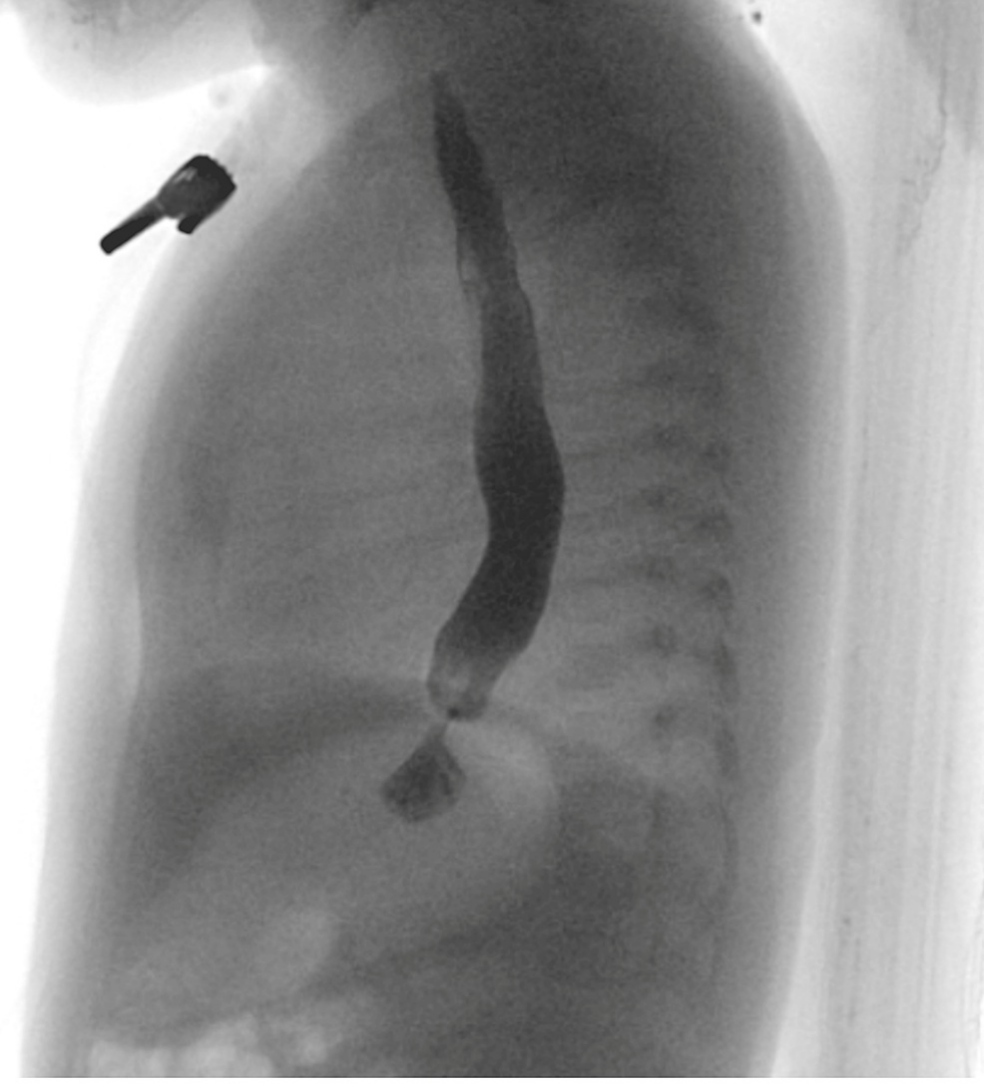
Upper GI tract showing severe gastroesophageal reflux GI: Gastrointestinal

**Figure 2:**
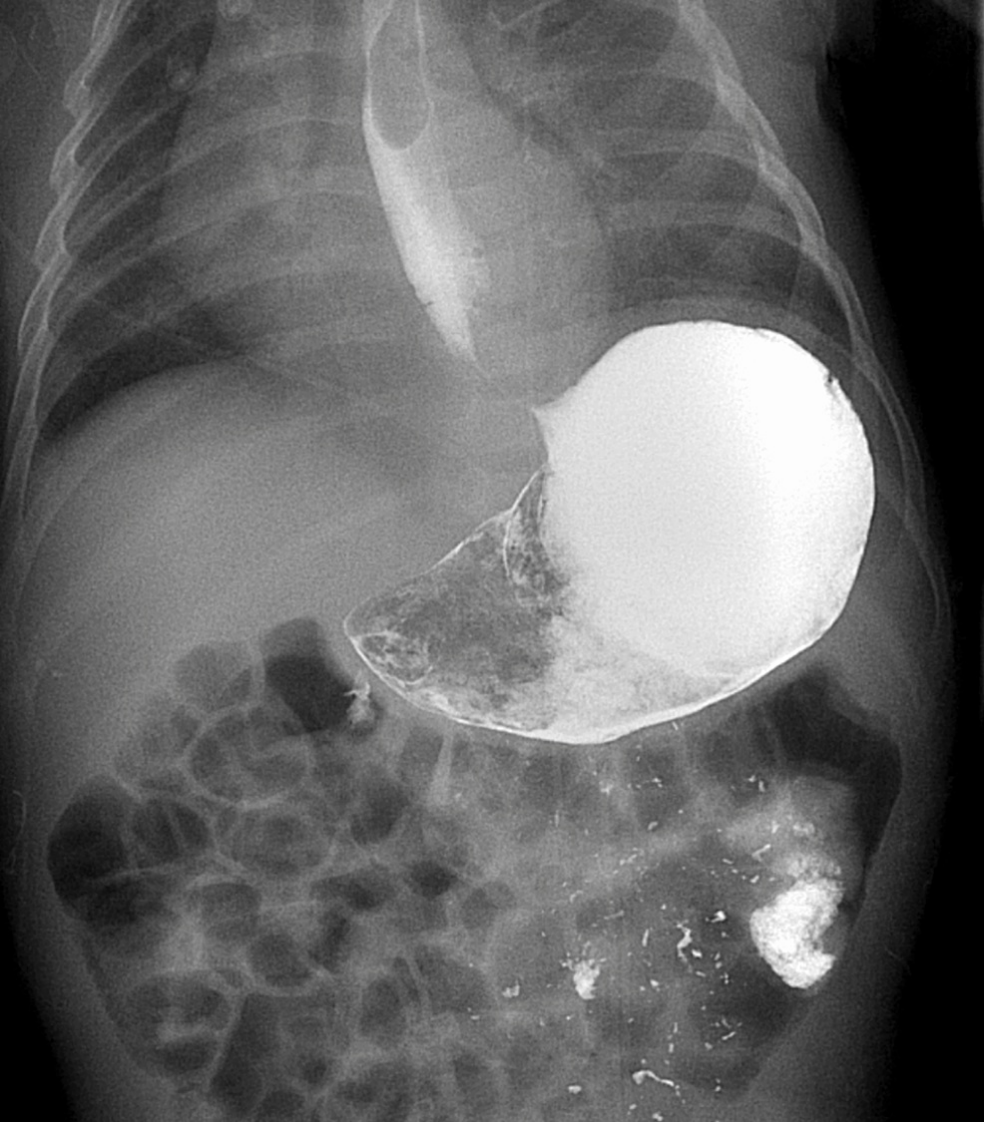
Upper GI tract showing significant narrowing of pyloric channel with very minimal gastric emptying consistent with gastric outlet obstruction GI: Gastrointestinal

**Figure 3:**
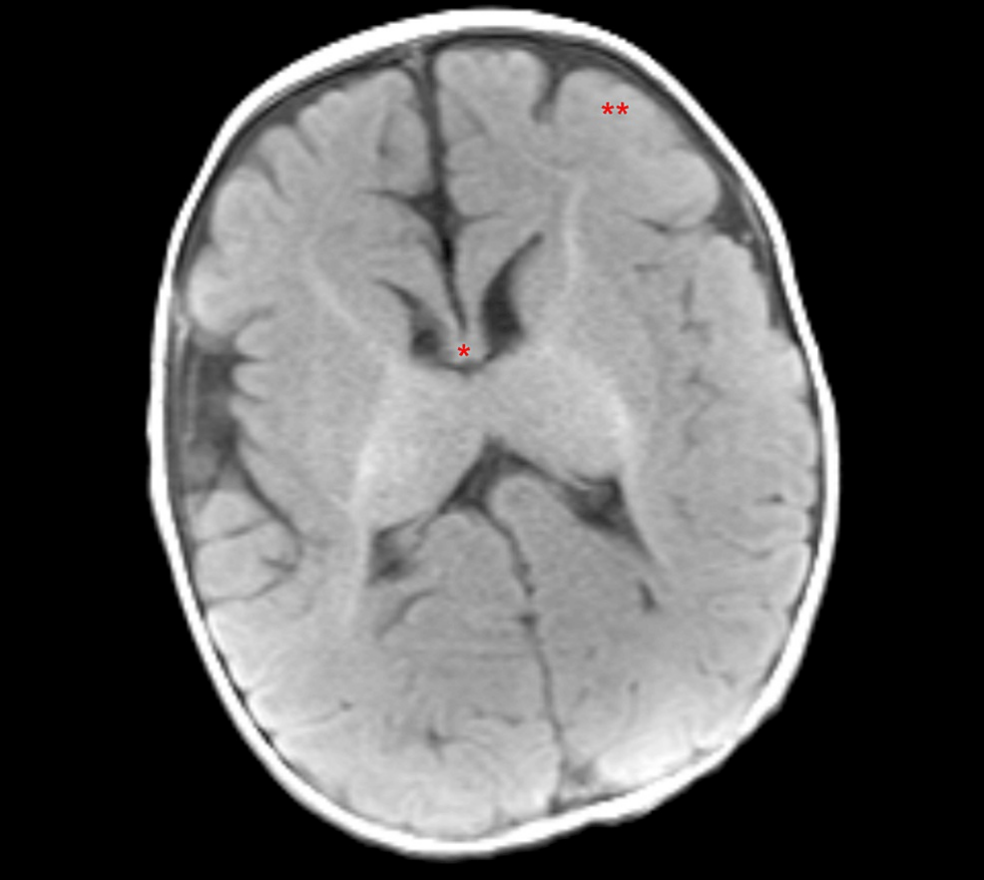
Patient's brain MRI showing corpus callosum hypoplasia and cortical malformations in the frontal-parietal lobes, suggestive of pachygyria * Corpus callosum hypoplasia ** Pachygyria

He was subsequently transferred from the gastroenterology clinic to the emergency department and underwent extensive interdisciplinary workup between gastroenterology, surgery, pulmonology, and ENT. He was diagnosed with gastric outlet obstruction following an upper gastrointestinal series (UGI) (Figures 1, 2). The patient underwent a pyloroplasty. He also underwent a brain MRI, which showed corpus callosum hypoplasia and cortical malformations in the frontal-parietal lobes, suggestive of pachygyria (Figure 3). The patient was evaluated over two additional months. He returned to the clinic, now at four months of age, to follow up status/post pyloroplasty. The family reported that he had experienced poor progression in developmental milestones, most notably secondary to profound hypotonia. Additionally, over the last few months, physical therapy and feeding therapy have been incorporated. Due to the patient’s hypotonia and inability to support his head, he was struggling to feed by mouth, consistent with oropharyngeal motor dysfunction. In addition to the failure to thrive, global developmental delay and hypotonia, the patient also experienced associated symptoms of ichthyosis and on examination, the patient had roving eye movements. It was recommended the family obtain genetic testing, uncovering a diagnosis of CEDNIK syndrome, with the parents being carriers for the disease. The patient’s genetic report yielded a region of homozygosity (13.7 Mb) at 2p25.2p24.2(4951900_18695114).

The patient's treatment remained focused on feeding tolerance. Achieving appropriate weight gain is essential to maintain a healthy growth as well as progress to meet developmental milestones. The incorporation of feeding and physical therapies with focused treatment on oropharyngeal control have shown positive results, however, transition to pureed or solid foods is not indicated at this time due to inability to support his head on his own.

## Discussion

As initially described in 2005, CEDNIK syndrome is known to be an autosomal recessive and genetically mapped to the 22q11.2 gene [3] with disease manifestation in the homozygous haplotype [3]. This homozygous haplotype spanned four Mb and has been found to be shared amongst all afflicted patients, and are absent or carried in a heterozygous state, by otherwise healthy family members [3]. Additionally, in the 19 patients reported with CEDNIK syndrome, there is not a clear genotype-phenotype correlation [2]. The SNAP29 protein can further be described as encoded by a 5-exon gene, which is involved with intracellular membrane structures, playing a role in membrane trafficking processes [4]. SNAP29 is then involved with binding multiple syntaxins and aiding in vesicle transport, recycling, and fusion, via its two soluble N-ethylmaleimide-sensitive fusion protein attachment protein receptor (SNARE) domains [4]. Through the SNARE protein, SNAP29 is also able to affect autophagy, cell motility, and ciliogenesis [4].

The significance of understanding the pathophysiology pertains to its correlation with proposed disease mechanisms. The role of SNARE domains is tied to the brain during development, with axonal growth, synaptogenesis, and neurotransmission [3]. In terms of neurotransmitter regulation, SNAP29 is involved through attenuation of the recycling of fusion machinery, thus slowing synaptic vesicle turnover [3]. SNARE complex formation and dissociation and recycling are vital for synaptic transmission, which can serve as an explanation for why patients develop the aforementioned neurologic sequelae [3]. SNARE proteins also play a significant role in the development of the retina, thus suggesting why patients tend to develop roving eye movements, which are a common initial presenting sign in the disease process [3].

Patients, such as the one described in our case, also develop dermal hyperkeratosis as well as ichthyosis. The pathophysiology undermining the cutaneous aspect of the syndrome is likely attributed to abnormal lamellar granule maturation and secretion [3]. The way this process works in normal skin is through release of lipids and proteases below the stratum corneum, which are vital in mediation of the epidermal barrier and with desmosome desquamation [3]. In CEDNIK syndrome, glucosylceramide and kallikrein products containing granules are retained in the stratum corneum, preventing desmosome detachment and leading to pathologic barrier formation, causing retention hyperkeratosis [3].

The seven cases initially reported during the primary investigation of CEDNIK syndrome showed similar disease progression amongst the patients (Table 1) [3]. Initially, patients had an uneventful gestation without abnormalities in APGAR score and birth weight [3]. In the ensuing four months of life, symptoms such as roving eye movements, poor head and trunk control, and failure to thrive were seen [3]. Between 5 months and 11 months of life in these cases, the dermatologic manifestations ensued [3]. By the age of 8-15 months of life, psychomotor slowing became prominent, as the ability to reach developmental milestones, such as sitting and walking, stalled. In our case, the patient too had an uneventful birth, however at two months, he developed failure to thrive, GERD, and stridor. He also developed gastric outlet obstruction secondary to pyloric stenosis requiring pyloroplasty. The neurologic timelines in the previously described cases also aligned with our case as the patient also had roving eye movements, along with poor head and trunk control, attributed to profound hypotonia by this time.

**Table 1:** Established timeline of disease progression Timeline of clinical manifestations in the initial presenting study [3]. * Indicates manifestation is also seen in our four month old patient. Note: The patient in our case developed gastric outlet obstruction requiring pyloroplasty.

Birth	Birth-4 months	5-11 months	8-15 months
Uneventful Gestation*	Failure to Thrive*	Ichthyosis*	Unable to sit unsupported
Normal APGAR*	Roving eye movements*	Keratoderma*	Unable to walk
Normal Birthweight*	Poor Head and Trunk Control*	-	Psychomotor slowing

Additionally, CEDNIK syndrome may present with pathognomonic brain MRI findings. These typical findings include hypoplasia or dysplasia of the corpus callosum, as well as polymicrogyria/pachygyria [2]. Brain stem malformations have also been documented, which are evident by elongated pons and shortened medulla [1]. In our case, the patient’s MRI was consistent with previously documented findings, as his corpus callosum was markedly hypoplastic. Cortical malformations were also seen in the case of our patient, which were present in the bilateral cerebral hemispheres, particularly in the frontal and parietal lobes - suggestive of pachygyria. His brain MRI also showed normal ventricular size without hydrocephalus and was without evidence of oedema or intracranial hemorrhage. In the 2021 Cohort study of six new patients with CEDNIK syndrome, three of them had abnormal EEGs [2]. At age four, patient 1 in this cohort developed seizures that, on EEG, showed multifocal epileptiform discharges [2]. Patient 2 in that cohort had a seizure at the age of three years with a normal EEG [2]. Lastly, patient 5 in their cohort developed seizures at four months, with multifocal epileptiform changes on EEG [2]. Our patient, at four months of age, had not experienced seizure like activity, and his EEG was unremarkable. In this same 2021 cohort, of the six patients, two of them experienced gastroesophageal reflux disease, with one of the patients having gastrointestinal dysmotility that was treated with domperidone [2]. The patient in our case developed pyloric stenosis resulting in gastric outlet obstruction at two months of age, confirmed by upper GI series, that required pyloroplasty. Upon review of the seven patients from the initial study in 2005, as well as the 2021 six patient cohort, none of the patients were reported to have gastric outlet obstructions [2, 3]. This finding is likely incidental as firstborn males, such as the patient in this case, have a statistically significant higher probability of developing pyloric stenosis [5]. Although this is likely an idiopathic finding, it has contributed to the patient’s failure to thrive. In the 2005 initial seven cases that were well studied, progression into adolescence yielded three male patients succumbing to aspiration pneumonia between the ages of 5 and 12, with the other four patients living between 5 and 13 years with previously described symptoms [3]. 

## Conclusions

CEDNIK syndrome is an extremely rare genetic disease with only a few cases reported worldwide. The combination of being new in discovery coupled with low incidence has led to uncertainty surrounding prognosis. As CEDNIK syndrome involves an assortment of systemic problems, care coordination between specialists is essential to disease management. Treatment options have not been well documented. However, in our case, the patient has worked with feeding and physical therapies with the aim of improving oropharyngeal function. The goal of these therapies is to improve oropharyngeal function to help maintain a healthy growth. As a major cause of death in CEDNIK syndrome's patients is failure to thrive, the goal to achieve appropriate weight gain should take precedence.

Additionally, patients may require a gastrostomy tube to allow sufficient caloric intake. As aspiration pneumonia has been a significant contributing factor to mortality, improving oropharyngeal coordination can help mitigate some risks to its development. The patient, in this case, has seen improvement in his feeding since starting therapy. However, transition to pureed or solid foods is not indicated at this time due to his inability to support his head on his own. Further research is warranted to determine the long-term prognosis of this syndrome as well as treatment options.
